# A Combined Genomics and Phenomics Approach is Needed to Boost Breeding in Sugarcane

**DOI:** 10.34133/plantphenomics.0074

**Published:** 2023-07-14

**Authors:** Ting Luo, Xiaoyan Liu, Prakash Lakshmanan

**Affiliations:** ^1^ Key Laboratory of Sugarcane Biotechnology and Genetic Improvement (Guangxi), Ministry of Agriculture and Rural Affairs, Sugarcane Research Institute, Guangxi Academy of Agricultural Sciences, Guangxi Key Laboratory of Sugarcane Genetic Improvement, Guangxi Academy of Agricultural Sciences, Nanning 530007, China.; ^2^ Interdisciplinary Research Center for Agriculture Green Development in Yangtze River Basin, College of Resources and Environment, Southwest University, Chongqing 400716, China.; ^3^ Queensland Alliance for Agriculture and Food Innovation, University of Queensland, St Lucia 4067, QLD, Australia.

Sugarcane is a major food and bioenergy crop globally. It produces ~80% of sugar consumed worldwide, with Brazil and India together accounting for 61% of world sugarcane production in 2021 [1]. Globally, sugarcane is the 5th largest crop by production value and acreage, and it is also the second largest bioenergy crop [1,2]. Modern sugarcane is an interspecific hybrid (*Saccharum* species hybrid) of wild progenitor species *Saccharum officinarum* (2*n* = 80; *x* = 10) and *Saccharum spontaneum* (2*n* = 40 to 130; *x* = 8) [3]. This genetically complex polyploid crop with varied chromosome numbers (100 to 130) has one of the largest genomes (~10 kb) among plants, making sugarcane breeding considerably slow and challenging.

Sugarcane breeding involves visual clonal selection combined with manual screening for cane stalk weight and cane sugar content through a 10- to 12-year-long multistage selection scheme with disease screening incorporated toward the end of the selection program. Globally, the rate of sugarcane yield improvement realized at commercial crop production level through breeding in recent decades remains considerably lower than that of other major crops, and in some breeding programs, genetic gain appears to have plateaued [1].

Long breeding cycle, practical difficulties for extensive phenotyping of breeding populations, low narrow-sense heritability of economically important traits, large complex polyploid genome with high heterozygosity, and genotype–environment–management interaction effects have been attributed to low rate of genetic gain. More specifically, the high biomass of sugarcane plants makes accurate detailed phenotyping logistically very challenging, which compromises selection accuracy. This is particularly so in the early stages of selection confounded by large interplot competition effects caused by small single- or 2-row plots [4]. Thus, accurate, cost-effective, and high-throughput phenotyping offers an excellent opportunity for more precise estimation of true yield potential of sugarcane clones in breeding trials, a major bottleneck for fast-tracking sugarcane improvement [5].

Recognizing the persisting slow yield improvement from sugarcane breeding and the accelerated genetic gains realized through molecular marker-assisted selection (MAS) in various other crops [6,7], some of the leading sugarcane industries invested substantial resources for sugarcane genome sequencing and MAS in the past 3 decades [8]. Over this period, sugarcane DNA marker systems have gradually evolved from the initial hybridization-based [9] to the current DNA-sequence-derived single-nucleotide polymorphism (SNP) markers, facilitated by high-throughput next-generation sequencing technologies [8]. The rapid advancements in DNA sequencing and marker technologies led to the creation of genotyping systems for whole-genome profiling, such as genomic selection (GS), which further strengthened marker discovery and marker-trait association studies. GS is a robust genotyping method capable of using large number of trait-linked DNA makers (e.g., SNP markers) spread across the whole genome and provides a more robust estimation of the genetic merit of a clone (for economically important traits) than previously achieved. Multiple independent studies found that it can be used as an effective high-throughput genetic screen for selecting elite sugarcane clones in breeding programs [8]. Using SNP markers, Deomano et al. [10], Yadav et al. [11], and, more recently, O’Connell et al. [12] proved the relative advantages of GS over conventional selection for cane yield, sugar content, and disease resistance. However, despite the long history of molecular marker discovery, application of MAS, including GS, in sugarcane breeding is yet to be realized.

Reliable, accurate phenotyping of large genetically diverse genotyped populations on a regular basis is required to implement GS in sugarcane breeding. While experimental data and modeling have shown the predictive power (estimation of clone genetic merit) and potential variety development application of GS in sugarcane, its success in breeding for complex quantitative traits such as cane yield and sugar content, which are predominantly controlled by nonadditive genetic effects (i.e., interactions among genes or alleles that are not readily passed on from parents to progeny) [11], depends on, at least in part, accurate, high-throughput, and cost-effective phenotyping. High-throughput phenomics thus will greatly reduce the cost and increase the efficiency of creating reliable GS training sets and phenotypic data required for building statistically robust clone performance prediction models needed for selection [8,10]. Further, on an ongoing basis, these GS prediction models need to be refined with the inevitable change in genetic background of breeding populations and the target production environments with diverse agroecological conditions.

Parallel to genomics technologies, sensor-based phenotyping advanced relatively rapidly in the past 2 decades. High-throughput phenotyping has been used extensively for improving crop agronomy and yield forecasting in many crops including sugarcane [13,14]. However, application of phenomics in commercial crop breeding is in its infancy, despite the fact that phenotyping forms the core of breeding and that it is very resource-intense. Interestingly, at least in grain crops, much of the research driving phenotyping innovations that are likely to transform breeding comes from the application of phenomics to unravel the hitherto intractable aspects of crop physiology and plant development [15]. For instance, phenomics-assisted yield prediction using indirect traits has been established in cereals [16], and its potential breeding applications have been emphasized in recent reviews [15]. Practically, a large number of plant growth, developmental, physiological, and leaf spectral attributes as well as certain tissue chemical parameters associated with biomass accumulation and crop yield can be quantified using high-throughput phenotyping platforms, some of which are now commercially available. However, not all biomass- or yield-linked indirect traits are suitable for clone selection in breeding. Ideally, indirect traits appropriate for breeding application must have at least some of the attributes shown in Figure, including high genetic correlation with and contribution to yield, directly linked to key physiological or developmental processes controlling growth and/or yield, high heritability, large genetic variation, and amenable to high-throughput phenotyping. Thus, selection systems incorporating appropriately chosen indirect yield traits may facilitate greater genetic gains, and economic return as heritability of such traits can be higher than yield itself [5].

Practically many of the previously intractable indirect yield-related traits are now readily amenable to high-throughput phenotyping. For instance, high-throughput phenotyping of various features of canopy development and canopy architecture, leaf light interception, canopy reflectance, plant height, leaf area, crop growth rate, canopy conductance, canopy temperature, leaf senescence, and photosynthesis has been reported [15]. In addition, many of these surrogate traits have been successfully used to develop prediction models for cereal crop biomass, water use, radiation use efficiency, crop photosynthesis, nitrogen accumulation, and yield [15,17]. Although not advanced to the extent as seen in grain crops, substantial efforts on application of phenomics in sugarcane agriculture have been reported (Table) [5,14,18]. Sugarcane is an ideal crop for field phenomics. Its large, high biomass stem is the harvested plant part, takes 12 to 22 months to mature, grown mainly in rain-fed marginal lands, and, thus, experiences recurrent climatic extremes, especially water deficit, widespread incidence of pests and diseases, and, above all, there is a real need for accurate, high-resolution, high-throughput phenotyping to accelerate genetic gain from sugarcane breeding.

Increasing stem biomass and cane sugar content are the ultimate objectives of sugarcane breeding for sugar production, while boosting total above-ground biomass with increased fiber content forms the target for energy cane breeding. Sugarcane growth is highly responsive to N and water supply, and one of the initial and common applications of sugarcane aerial field phenomics was tracking leaf N for N fertilization management [14]. While this agronomic application becomes very popular, its value in breeding comes from the positive relationship of leaf N content, leaf chlorophyll content, and sugarcane radiation use efficiency, which, in turn, determines biomass production. Similarly, research conducted by Basnayake et al. [19] using genetically broad-based sugarcane populations showed that canopy conductance and its surrogate trait canopy temperature have strong genetic correlation with cane yield across a range of growing conditions and that canopy temperature could be used as a predictor of sugarcane yield. Building on these studies, Natarajan et al. [5] constructed an indirect selection index for yield prediction using a multitude of secondary yield traits and showed its superiority over conventional clone selection under single-row and multirow (pure stand) conditions in large commercial sugarcane breeding trials in Australia. This pioneering study clearly established the potential of high-throughput field phenotyping of yield-associated indirect traits for improving clonal selections in sugarcane breeding.

The ongoing progress and investments in next-generation sequencing technologies, genomics, marker discovery, marker systems development, sequencing the genome of sugarcane and related species, bioinformatics and big data analytics, and investments in implementing GS and phenomics in some commercial sugarcane breeding programs are suggesting that MAS and high-throughput field phenomics will become an integral part of sugarcane breeding in at least in large sugarcane industries. Accelerated yield gain from GS-assisted sugarcane selection is very evident [10–12]. Integrating field phenomics into GS-assisted sugarcane breeding will further boost the realized rate of genetic gain and yield improvement. Drought tolerant maize developed for US maize industry forms the first example of discovery to product delivery (drought tolerant maize) from a breeding program integrating genomics, phenomics, and trait and crop modeling [20]. The remarkable progress made in developing sugarcane GS system [8,10–12], high-throughput phenomics-assisted screening of sugarcane for various crop performance traits [5,18], and sugarcane trait/crop modeling [2] now presents a unique opportunity to bring together and exploit these powerful tools and technologies to transform sugarcane breeding for the future.

**Figure.**
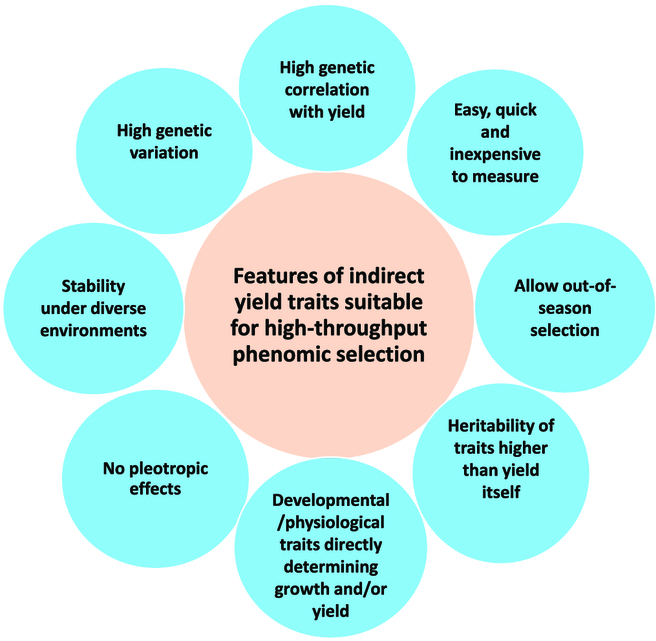
Desirable attributes of yield-associated indirect selection traits with potential for accelerated genetic improvement of sugarcane through breeding. A potential trait used for selection may have one or multiple attributes shown in this figure.

**Table.** Traits amenable to high-throughput phenotyping (HTP) and their potential for coapplication with GS for sugarcane breeding. The applicability of HTP and /or GS for these traits has been demonstrated in sugarcane experimentally^a^.

**Traits**		**Genotyping technology**	**Marker systems** ^**b**^	**Sensor systems**	**Application**	**Genomics-phenomics integration potential**	**Current status**
**Genomics**	**Phenomics**						
Cane yield	Cane yield	AAS array, DArT, and GBS	SNP markers and DArT markers	Multidimensional RGB, LiDAR, and multispectral imaging	Crop yield improvement, crop growth assessment	High	Limited institutional capacity and marker cost still limit wider application; targeted implementation efforts underway
Sugar content	Sugar content	AAS array, DArT, and GBS	SNP markers and DArT markers	NIR	Crop yield improvement	Medium to high	As above, hyperspectral imaging research for HTP
Fiber content	Fiber content	AAS array, DArT, and GBS	SNP markers and DArT markers	NIR	Stalk quality and energy cane	Medium to high	Moderate high-throughput currently
Stalk height, stalk number, and biomass	Stalk height, stalk number, and biomass	AAS array, DArT, and GBS	SNP markers and DArT markers	LiDAR and multidimensional RGB	Crop yield, stalk weight, rationability, and crop establishment	Medium to high	Used for crop growth monitoring and crop management
	Canopy cover and canopy architecture			LiDAR and multidimensional RGB	Canopy filling, crop gap, light interception, and water use	Limited genomics research	Extensively used for crop growth modeling and crop monitoring
Brown rust	Brown rust	AAS array and DArT	*Bru* gene	RGB and multispectral imaging	Brown rust resistance	High	Currently limited implementation in breeding programs
Orange rust, smut, and red rot		AAS array	SNP markers		Diseases resistance	Limited phenomics research	Application of smut markers for GS underway
Flowering	Flowering	AAS array	SNP markers	RGB and multispectral imaging	Flowering time and flowering propensity	High	Breeding application not reported
	Canopy conductance and canopy temperature			IR temperature sensors and thermal camera	Crop water use and thermal and water stress tolerance	Little genomics research on this trait	A valuable trait for breeding sugarcane for water-limited environments
	Early-stage crop vigor			RGB and multispectral imaging	Early-stage clone selection in breeding	Little genomics research on this trait	Value of this trait for improving early-stage clone selection demonstrated

^a^ References for the traits listed in this table can be found in [5,8,10,11,13–15,18,19].

^b^ These are the most frequently tested sugarcane DNA marker systems, but other polymerase-chain-reaction-based and single-gene marker systems with high-throughput screening potential have been reported for sugarcane by different groups.

AAS array, Affimetrix Axiom SNP array; DArT, Diversity Array Technology; GBS, genotyping by sequencing; RGB, red, green and blue; LiDAR, light detection and ranging; NIR, near-infrared.
